# Supplementary material for: Social and Behavioral Predictors of Adolescents’ Positive Attitude towards Life and Self
Source: Int J Environ Res Public Health. 2019 Nov 11;16(22):4404. doi: 10.3390/ijerph16224404 (PMC6888235; doi:10.3390/ijerph16224404)
Supplement: Supplementary file 1 [file ijerph-16-04404-s001.pdf]

Table S1. Correlations between the variables in the study group.

		1.	2.	3.	4.	5.	6.	7.	8.	9.	10.	11.	12.
1. Positive Attitude Scale (PAS)		1											
2. Family Affluence Scale (FAS)	r	0.077	1										
	p	0.000											
3. Physical Activity (MVPA)	r	0.174	0.065	1									
	p	0.000	0.001										
4. Tobacco smoking	r	-0.092	0.013	-0.046	1								
	p	0.000	0.487	0.013									
5. Alcohol drinking	r	-0.082	0.040	-0.001	0.520	1							
	p	0.000	0.032	0.961	0.000								
6. Being drunk	r	-0.070	0.028	0.017	0.444	0.686	1						
	p	0.000	0.143	0.359	0.000	0.000							
7. Cannabis use	r	-0.050	0.028	0.024	0.324	0.290	0.350	1					
	p	0.008	0.143	0.193	0.000	0.000	0.000						
8. Academic achievements	r	0.278	0.102	0.064	-0.250	-0.205	-0.188	-0.135	1				
	p	0.000	0.000	0.001	0.000	0.000	0.000	0.000					
9. School stress	r	-0.249	-0.054	-0.048	0.146	0.127	0.124	0.062	-0.340	1			
	p	0.000	0.004	0.009	0.000	0.000	0.000	0.001	0.000				
10. Family communication	r	0.366	0.172	0.065	-0.158	-0.148	-0.129	-0.121	0.175	-0.236	1		
	p	0.000	0.000	0.001	0.000	0.000	0.000	0.000	0.000	0.000			
11. Peers communication	r	0.194	0.054	0.003	0.081	0.041	0.019	0.009	0.032	0.017	0.189	1	
	p	0.000	0.004	0.856	0.000	0.029	0.299	0.645	0.089	0.354	0.000		
12. Neighborhood social capital	r	0.232	0.054	0.121	-0.025	-0.026	-0.004	-0.014	0.071	-0.074	0.252	0.156	1
	p	0.000	0.005	0.000	0.193	0.177	0.832	0.461	0.000	0.000	0.000	0.000	

Note: *r-Pearson correlation; p-significance level
